# Synesthesia, Sensory-Motor Contingency, and Semantic Emulation: How Swimming Style-Color Synesthesia Challenges the Traditional View of Synesthesia

**DOI:** 10.3389/fpsyg.2012.00279

**Published:** 2012-08-22

**Authors:** Aleksandra Mroczko-Wąsowicz, Markus Werning

**Affiliations:** ^1^Institute of Philosophy of Mind and Cognition, National Yang-Ming UniversityTaipei, Taiwan; ^2^Department of Philosophy II, Ruhr University of BochumBochum, Germany

**Keywords:** synesthesia, sensory-motor contingency, simulation, hyperbinding, emulative semantics, frames, embodied cognition, mirror neuron system

## Abstract

Synesthesia is traditionally regarded as a phenomenon in which an additional non-standard phenomenal experience occurs consistently in response to ordinary stimulation applied to the same or another modality. Recent studies suggest an important role of semantic representations in the induction of synesthesia. In the present proposal we try to link the empirically grounded theory of sensory-motor contingency and mirror system based embodied simulation/emulation to newly discovered cases of swimming style-color synesthesia. In the latter color experiences are evoked only by showing the synesthetes a picture of a swimming person or asking them to think about a given swimming style. Neural mechanisms of mirror systems seem to be involved here. It has been shown that for mirror-sensory synesthesia, such as mirror-touch or mirror-pain synesthesia (when visually presented tactile or noxious stimulation of others results in the projection of the tactile or pain experience onto oneself), concurrent experiences are caused by overactivity in the mirror neuron system responding to the specific observation. The comparison of different forms of synesthesia has the potential of challenging conventional thinking on this phenomenon and providing a more general, sensory-motor account of synesthesia encompassing cases driven by semantic or emulational rather than pure sensory or motor representations. Such an interpretation could include top-down associations, questioning the explanation in terms of hard-wired structural connectivity. In the paper the hypothesis is developed that the wide-ranging phenomenon of synesthesia might result from a process of hyperbinding between “too many” semantic attribute domains. This hypothesis is supplemented by some suggestions for an underlying neural mechanism.

## Introduction: Synesthesia

Even though we are equipped with similar sensory mechanisms and cognitive functions, the way we perceive the world around us differs between subjects. An attempt to realize these individual differences as well as the commonalities in epistemic abilities makes us more sensitive to the problem of understanding the human mind. One of such extraordinary phenomena is synesthesia in which the stimulation of one sensory or cognitive pathway (the inducer) elicits stable sensory experiences (the concurrent) in the same or another modality (Baron-Cohen and Harrison, 1997; Mattingley et al., 2001; Ramachandran and Hubbard, 2001a,b). Some synesthetes have color sensations (e.g., red) seeing an alphanumeric symbol (e.g., an “A” or a “2”), individuals with another kind of synesthesia perceive colored spatial moving patterns when hearing music or, more generally, sounds. Other forms of synesthesia relate tastes, smells, visual, or tactile experiences to one another in almost any combination. Potentially, a huge number of different forms of synesthesia exist. The prevalence of the phenomenon depends on the particular type of synesthetic association with grapheme-color synesthesia being the most frequent one (Cytowic and Wood, 1982; Mroczko-Wąsowicz and Nikolić, in press). The most characteristic feature of genuine synesthetic experiences is that they are non-acquired and involuntary conscious perception-like experiences which remain remarkably constant throughout the lifespan (Baron-Cohen et al., 1987). These consistent inducer-concurrent pairings may easily be differentiated from other, non-synesthetic associations. First, because they are elicited by a stimulus that normally is not associated with this additional, internally generated experience (Treisman, 2005). Second, although the associated experience, frequently color sensation, is as vivid and realistic as the veridical perception of the ink color, synesthetes always know which colors are ink and which are synesthetic. This exhibits the opacity of synesthetic phenomenal experiences (see, e.g., Metzinger, 1999). Simple consistent shape–color pairings that arise as a result of learning and memory associations are not likely to produce synesthesia. Such pairings occur often in our everyday life (e.g., the shape of a lemon is associated with the color yellow), but these matches do not convert into permanent synesthetic associations.

## Perspective: Cognitive Accounts in Synesthesia Research

One of the pressing questions in current research on synesthesia is the distinction between “low-” and “high-level” synesthesia, i.e., the problem of how to determine whether it is a sensory or semantic/conceptual phenomenon. At first, mostly the low-level explanation in terms of cross-talk of the senses has accumulated supporting evidence and dominated the debate. However, this appears to be merely a partial truth, since in many forms of synesthetic association only the concurrents exhibit purely perceptual features and inducers seem either to have a semantic nature or at least also to involve cognitive aspects, linking these types of synesthesia to high-level cognitive phenomena. Together with other authors (Dixon et al., 2006; Simner, 2007; Jürgens and Nikolić, 2012) we propose that a full account of the currently investigated phenomenon should transcend the traditional view. The traditional view is captured by the original compound “syn” + “aesthesia” (Greek for union of the senses) and takes synesthesia to be a solely perceptual phenomenon, such that a sensory stimulus of one modality elicits an additional perception in the same or another modality (e.g., Baron-Cohen and Harrison, 1997; Ramachandran and Hubbard, 2001b; Cytowic, 2002a,b). A more adequate understanding of the phenomenon should also take into account phenomena that do not merely involve sensations. For that purpose Nikolić et al. (2011) have coined the term *ideaesthesia*, *idea* + *aesthesia*, meaning sensing ideas, sensing concepts, and referring to the conceptual processing in synesthesia with perceptual concurrents and conceptual inducers joined together (see also Jürgens and Nikolić, 2012; Gómez Milán et al., in press). Such a phenomenon can be explained by high-level semantic mechanisms that guide the assignment and evocation of low-level sensory concurrents. Another analysis relates synesthesia to *the unity of cognition* and demonstrates that this phenomenon exhibits certain holistic epistemic ability integrating different mental faculties into a hypercoherently unified conscious experience (Mroczko-Wąsowicz, in press). In recent work Werning (2012) construes semantic representation as a form of non-symbolic compositional emulation, where the content objects, properties, and situations are emulated by brain mechanisms. Synesthesia very often involves inducers that are not strictly sensory, e.g., words, numbers, time units, musical notes, or personalities. Synesthetes may exhibit inducer-concurrent pairs with a cognitive representation of an abstract concept as an inducer and a sensory experience as a concurrent: Thinking about the number three – irrespectively of how the number is graphically represented, as “3,” “III,” or “. . .” – may produce a concrete color experience. Synesthetic associations are thus not merely cross-modal, but cross-domain, where the domains may not only involve the various sensual domains, but also the domains of bodily and emotional states as well as domains of abstract, conceptually represented entities like numbers or time units. Moreover, as we will highlight in this paper, synesthesia may even cross the motoric and sensory domains.

Evidence for this alternative perspective on synesthesia is getting stronger. Recent studies suggest an important role of semantic representations in the induction of synesthesia. The term “synesthetic conception” introduced by Grossenbacher and Lovelace (2001) accounts for the conceptual aspects involved in synesthesia. In the above mentioned time unit-space synesthesia (e.g., Smilek et al., 2007; Jarick et al., 2008; Mann et al., 2009) subjects experience units of time, typically hours, days of the week, and months, as being placed at specific spatial locations in reference to their body. Semantic aspects of synesthesia can furthermore be identified in the “tip of the tongue” phenomenon or generally in lexical-gustatory forms of synesthesia. Here the verbalization of the stimulus is not necessary for the sensation of taste and the activation of the respective concept is sufficient (Simner and Ward, 2006). Cases of synesthesia that transcend traditionally denoted sensory modalities can also be found in reading musical notes, calculating, imagining, or just thinking of a stimulus (Frith and Paulesu, 1997; Grossenbacher, 1997; Dixon et al., 2000, 2006; Ramachandran and Hubbard, 2001a; Cytowic, 2002a; Rich et al., 2005; Ward et al., 2006). Synesthesia is also prevalent as an intralinguistic phenomenon in so-called synesthetic metaphors: “loud colors,” “itching tunes,” “cold smell.” Here a concept from some sensory modality is used to modify a concept from another sensory modality. Interestingly, synesthetic metaphors can be communicated and are understood across subjects simply on the basis of shared semantic knowledge and without the use of the relevant sensory information channels (Williams, 1976; Yu, 2003; Werning et al., 2006). Also, a substitution of familiar graphemes with exotic, newly learned ones with the same meaning – the letter “A,” e.g., now being replaced by a Glagolitic grapheme – can result in a transfer of synesthetic color experiences in less than 10 minutes of training (Mroczko et al., 2009). Therefore, synesthesia seems to rely essentially on a certain interpretation of the stimulus and the meaning that it has for the subject. To account for these phenomena we have to assume that the meaning of the inducing stimulus for the subject has to be read off before the concurrent experience can occur. We can no longer maintain that the synesthetic association is caused solely by low-level hard-wired preexisting connections between sensory areas (Dixon et al., 2000; Nikolić et al., 2011; Jürgens and Nikolić, 2012).

## Theory and Hypothesis: Sensory-Motor Contingency, Emulation, and Synesthesia

In philosophy of mind and cognitive neuroscience advocates of embodied cognition propose that many features of cognition are causally or constitutively associated with the physical body and bodily actions of an agent. According to this line of thinking the sensorimotor account of perception (known also as enactivism) states that our senses mediated by different forms of sensory-motor contingency explore the environment. The mind is constituted by the sensory-motor contingency between the agent and the world (Noë, 2005). The sensorimotor theory is a high-level cognitive model of conscious experience in which sensory experience results through the subject’s cognitive access to a sensory-motor skill (O’Regan and Noë, 2001a). According to this model, consciousness arises from representation of interactions between organism and environment such as sensory changes induced by different motor actions, i.e., sensory-motor contingencies. Thus, phenomenal differences between various perceptual experiences can be accounted for by different actions when using different sensory modalities; transformations in qualitative experience may well be explained in terms of a dynamic model of interdependence between sensory or semantic inputs and embodied activity (Hurley and Noë, 2003). Hence, a distinctive pattern of sensory-motor contingency conditions the subject to act in a manner such that the ways things appear to the subject are affected. This results in a matching between bodily and environmental features (O’Regan and Noë, 2001a,b; O’Regan et al., 2006). The theory is supported by empirical findings regarding effective sensory substitution, sensory-motor adaptation for color perception or for touch as found for instance in the rubber hand illusion and mirror therapy reducing phantom limb pain (Ramachandran and Rogers-Ramachandran, 1996; Botvinick and Cohen, 1998; Bompas and O’Regan, 2006; Proulx and Störig, 2006). Another sensory-motor contingency may be observed in the matching between different modalities and domains. The theory emphasizing these issues, called embodied social cognition or emulation/simulation theory, explains the phenomenon of intersubjectivity as intercorporeity or visuo-tactile matching in various positive symptoms employing mirror neuron systems and other mirroring mechanisms in our brain (Gallese and Goldman, 1998; Rizzolatti and Sinigaglia, 2010). Mirroring others’ experiences requires a mapping between the self and others. This, indeed, may involve some of the brain mechanisms underlying social competencies. Mirror systems may have evolved as an adaptation for interpersonal understanding (Gallese and Goldman, 1998). They refer to cortical areas that respond both to observing another person’s state and being in that same state oneself (Gallese, 2003). Mirror systems have been reported in humans not only for actions (Rizzolatti et al., 1996), but also for emotions (Bastiaansen et al., 2009; disgust – Wicker et al., 2003; facial expressions – Carr et al., 2003), and sensations (pain – Avenanti et al., 2005; touch – Blakemore et al., 2005). Hence, these mechanisms map the sensory representations of the sensation, emotion, or action of an observed person onto the perceiver’s own somatosensory, viscero-motor, or motor representations of the sensation, emotion, or action. Such a mapping enables the observer of another person’s sensation, emotion, or action, to feel as if he were experiencing that sensation or emotion or performing that action himself. The mirroring mechanisms represent sub-personal instantiations of embodied emulation, i.e., respective neural activations for brain-body states associated with sensations, emotions, and actions evoked in the observer while observing social stimuli as if he were undergoing a similar experience.

Generally speaking, an emulator is a device that mimics the state transitions of a target system due to some structural mapping (total or partial isomorphism, homomorphism, etc.) between the states and transitions of the device and the states and transitions of the target system. Emulators may thus have representational content due to their structure and their transitional performance. Being dynamical systems with representational powers, they may also serve as forward models and generate predictions (Grush, 2004; Bartels, 2006). Emulation is a subcategory of simulation, but typically contrasts with high-level simulation which might, e.g., be based on global mathematical equations. In the cognitive science literature the notions “emulation” and “simulation” are often used interchangeably.

Embodied social emulations exhibit the relations that our brain bears to other persons by mapping others’ sensations and emotions onto our cerebral somatosensory and viscero-motor states, and others’ actions onto our cortical motor states (Gallese and Metzinger, 2003; Gallese, 2007; Gallese and Sinigaglia, 2011). These brain states are themselves representational and represent features, evaluations, and affordances of objects, events, and situations in the world as well as states of one’s body and potential actions resulting therefrom. Emulation plays a central role in acquiring knowledge about our environment (Gordon, 1986, 1995; Goldman, 1989, 1992; Metzinger and Gallese, 2003; Thomas et al., 2006). Effective perception or action requires the capacity of emulation in order to predict impending sensory events or consequences of action (Grush, 2004). Emulation is not restricted to the somatosensory or motor domain. In the theory of emulative semantics (Werning, 2012), emulations are interpreted as semantic values that can be linked to each other and thus become constitutive for concepts (see below).

Neural mechanisms of mirror systems seem to be involved in synesthesia. It is particularly vivid in forms of mirror-sensory synesthesia, e.g., mirror-touch or mirror-pain synesthesia (Blakemore et al., 2005; Banissy and Ward, 2007; Fitzgibbon et al., 2010; Keysers et al., 2010). This implies that many aspects of everyday social cognition can be conceptualized as synesthesia-like processing. The already illustrated phenomenon of sensory-motor contingency recalls other perceptual phenomena and mental processes in human cognition supported by multisensory, sensory-motor, or cross-activation mechanisms similar to those in synesthesia. In the present paper, and especially in this section, we want to point out the omnipresence of synesthesia-like processing and the relevance of an emulation theory of cognition in explaining such experiences. The reason for doing this is motivated by numerous studies on different multimodal, cross-activation phenomena emphasizing the inductive role of sensory-motor processing and semantic representations of the stimulus, such as mirroring one’s own body or self and projecting it outside the body via the imposition of multisensory conflicts using congruent and incongruent visuo-tactile inputs (Metzinger, 2005; Lenggenhager et al., 2007; Ionta et al., 2011; Ferri et al., 2012), autoscopic symptoms that occur when patients hallucinate their mirror image (Zamboni et al., 2005), undergo out-of-body experiences (Blanke and Metzinger, 2009), double delusions (Brugger, 2002), or feel the sense of presence in the widowhood hallucinations (Rees, 1971).

Inter-modal analogies outside canonical synesthesia are quite universal. We can agree on such commonly shared associations like experiencing higher pitch as lighter and smaller. Comparable evidence of correspondences between different domains exist for vision and touch (see, e.g., mirror-touch synesthesia, rubber hand and full body illusions) and is based on vicarious activation, activation of a brain region that is typically involved in processing the observer’s own actions and sensations, but that is now activated by seeing similar actions or sensations in another person. The observation of touch has long been considered a solely visual event. However, recent studies suggest the involvement of vicarious activations in somatosensation, i.e., that a somatosensory component is also activated in the observer. Observed touch is processed in visual parts of the brain and in somatosensory areas (Ebisch et al., 2008, 2011; Keysers et al., 2010). Watching another person being touched usually activates a neural circuit similar to that of the actual touch. Only “similar” because at the neural level the overlap is not total between one’s own experiences and the experiences observed in someone else. In the former case both primary and secondary somatosensory cortices (SI and SII) are activated. However, while observing the touch only vicarious activation in the secondary somatosensory cortex occurs (Keysers et al., 2010). Despite this vicarious SII activation, in daily life we are not confused about who is being touched. This is because the primary somatosensory cortex including the Brodmann’s area [BA] 3, is only recruited when we ourselves are being touched. This fact seems to shed some light on the functional foundation of the neural mechanisms underlying mirror-sensory synesthesia, explaining why certain people have conscious somatosensory experience during the observation of similar stimulation applied to another person. Here, the inducer is the “observed bodily touch,” rather than vision *per se*. Depending on whether this stimulus is applied to a synesthete or to a non-synesthete, an observation of touched body parts affects somatosensory activation and experiences to a different extent. A recent fMRI study with non-synesthetic healthy subjects shows that some brain regions involved in first-person sensory experiences appear to actively distinguish between self and other conditions, in the sense that they are positively modulated (activated) for first-person sensory experiences, but negatively modulated (deactivated) when observing these experiences in others. The specific pattern of negative modulation has been detected in the posterior insula for subjects watching affective social touch applied to other individuals. In contrast to this vicarious activation, a positive modulation in somatosensory regions has been found for the actual tactile experience. The posterior insula, embedded in a distributed network grounding a sense of the bodily self, seems to differentiate between self and others when affective experiences are implicated (Ebisch et al., 2011). The mentioned partial overlap and deactivation processes, may be responsible in the general population for not experiencing others’ experiences during social perception. They may explain why usually no synesthetic experiences occur when we simulate or mirror others’ bodily experiences. Alterations at this level could result in sensory experiences also when perceiving them in others as this is the case in experimentally or neurologically induced illusory situations (e.g., out-of-body experiences) or specific individuals such as mirror-sensory synesthetes.

Amputation or paralysis of a limb is frequently accompanied by tactile, painful, or motoric sensations in the location of the missing limb, a so-called phantom limb. Ramachandran and Rogers-Ramachandran (1996) used mirrors to create a duplicate image of the amputated arm based on a reflection of the patient’s existing arm. Observed movement or touch at the intact arm produced the identical proprioceptive sensations at the phantom limb. This synesthesia-like processing can be conceived of as a behaviorally induced form of synesthesia or a temporal sensory substitution. The phenomenon of synesthesia as well as others including cross-activation and mirroring mechanisms distinctly exhibit a multimodal integration and multisensory awareness of selfhood. Both of them seem to be necessary conditions for pre-reflective bodily self-consciousness.

The construction of one’s own body image requires an integration of multimodal information from such different sources as visual, haptic, and proprioceptive perception. Interactions between these different domains may also yield specific self-identification and embodiment illusions like the enfacement illusion (Sforza et al., 2010), the rubber hand illusion (Botvinick and Cohen, 1998) or the full body illusion induced on purpose in the lab with a virtual reality apparatus (Lenggenhager et al., 2007) or due to some neurological origin (Blanke et al., 2004). Studying the mentioned illusions and alterations of the body image based on a synesthesia-like processing caused by the interpersonal multisensory stimulation (felt touch and vision of touch) provides a promising tool for learning more about the neural representations of stimulated body parts as well as about the attribution, localization, and ownership of the bodily self (Tsakiris, 2008). Studies measuring visual-somatosensory spatial congruency effects for the full body and subjective changes in global bodily self-consciousness have shown that during incongruent trials, i.e., conflicting visuo-tactile input, tactile stimuli were mislocalized toward the observed body. Since no such tests have been done with mirror-somatosensory synesthetes, aside from studies revealing that these subjects have difficulties with distinguishing between actual and synesthetic touch (Banissy et al., 2009), future research should concentrate on discriminating differential brain activations related to cross-modal conflicts also in specific individuals (Aspell et al., 2009).

The difference between the above mentioned autoscopic phenomena and shared multisensory experiences in the mirror-(somato)sensory synesthesia such as mirror-touch or mirror-pain synesthesia is that in the former case the body is visually mirrored, and in the latter one – visually presented tactile or noxious stimulation of others results in a somatosensory activation in oneself, i.e., in the projection of the conscious tactile or pain experience onto the respective part of the observer’s body (Blakemore et al., 2005; Banissy et al., 2009). Mirror-sensory synesthetes feel on their bodies what observed people are feeling when they are really touched. Also in this form of synesthesia concurrents can be experienced even by simply thinking of the inducers, e.g., when imagining another person in pain (Fitzgibbon et al., 2012) or anticipating such an experience. It has been shown that anticipation of somatosensation can increase activation in the primary somatosensory cortex without actual stimulation (Carlsson et al., 2000). However, inducer-concurrent correspondences are not so individual here as in other forms. They are much more regular and dependent on the stimulus. For instance, the intensity of the felt touch is stronger when observed touch is applied to real bodies than to dummy bodies. This feature of synesthetic mirror-touch might be related to a more general differential susceptibility to the involvement of somatosensory and interoceptive cortices into embodied simulation found in fMRI studies with non-synesthetic healthy subjects (Ebisch et al., 2008, 2011). This suggests that top-down processes may also modulate the intensity of the synesthetic mirror-touch experience (Fitzgibbon et al., 2010; Holle et al., 2011). Different potential mechanisms of mirror-sensory synesthesia are discussed in the literature. The most widely proposed underlying mechanism is an over-activation of the mirror neuron system for somatosensation, significantly beyond the threshold for consciousness. Both mirror-touch synesthetes and non-synesthetes when experiencing and observing touch activate visuo-tactile mirroring mechanisms, i.e., similar brain areas (partially overlapping) in premotor and parietal regions, primary and secondary somatosensory cortices (Blakemore et al., 2005). Nevertheless, when observing touch on others, fMRI has revealed the difference in synesthetic as compared to non-synesthetic mirror-touch, namely a greater activation within somatosensory cortices (including BA 3) as well as bilateral activation of the anterior insula (Blakemore et al., 2005; Keysers et al., 2010). The latter has been related to self-awareness (Critchley et al., 2004) and processing one’s awareness of others (Lamm and Singer, 2010). The amount of neural activity within the insula has been shown to increase by directing attention to one’s own emotions when viewing affective stimuli (Straube and Miltner, 2011). Thus, the insular cortex is likely to be a candidate for modulating distinctions between the sources of one’s own and another’s tactile conscious experience within the mirror-touch system as well as an important component for constructing a self-model from sensory-motor emulation based mechanisms of social cognition.

These issues, however, have not been investigated thoroughly and systematically enough by neuroimaging studies. Since it seems that other mechanisms also may well be involved in the induction of synesthesia-like processing, other hypotheses regarding the functional basis of the mirror-sensory phenomenon have come up. Another potential underpinning considered in this context is associative learning and heightened empathy (Fitzgibbon et al., 2010). These processes are actually not mutually exclusive or contradictory to the hyperactivity of the mirror neuron system. Thus, their strengths should be pulled together in representing a more complex interplay of relevant processes providing an appropriate and full explanation of these cross-activated multisensory perceptions. The theory of mirror-sensory synesthesia as a learned association or a result of associative learning is encouraged by effects of sensory-motor modulation of mirror areas found in studies demonstrating that, e.g., ballet dancers exhibit an increased mirror neuron activity when observing ballet compared to dancers of other styles (Calvo-Merino et al., 2005). This may suggest the involvement of associative learning in the development of mirror systems. If so, mirror-sensory synesthesia would be the result of a learned association through sensory-motor experience mediated by mirroring mechanisms. This is not irreconcilable with the suggestion that mirror systems are over-activated in mirror-sensory synesthesia leading to a conscious somatic sensation. Studies on mirror-sensory experiences, especially those acquired in mirror-pain synesthesia in amputees, suggest that they may come about through disinhibition of systems involved in empathy for pain and as such they may be understood as a result of enhanced empathic capacity (Fitzgibbon et al., 2010). Visuo-tactile interaction as in mirror-touch synesthesia – and generally multisensory integration – enables the implementation of a number of social skills related to empathy and reading other minds, e.g., understanding intentions, feelings, and emotional states of other people. In the embodied simulation and sensory-motor approach to the theory of mind and social cognition it has been postulated that this interpersonal competence is not just an effect of rational reasoning about mental states of others. It seems to depend on a special human faculty of perspective taking by simulating other people’s aims and actions using mirror neuron systems (Gallese and Goldman, 1998; Gallese, 2007). Social interactions based on shared feelings and simulated experiences may be seen as a specific kind of synesthesia-like processing. Thus, sensory-motor exchange when observing others’ behavior may induce a concurrent motor reaction, e.g., automatic mimicking of someone’s facial expression (Dimberg, 1982) or sensory–sensory interactions when the empathic experience of someone’s pain elicits qualitatively similar somatosensory experience in the observer. Cross-modal associations exhibiting synesthesia-like processing are inbuilt both into veridical and illusory perceptions as well as into human (social) cognition. Integrating information from cognitive, motor, and perceptual domain, across different sensory modalities and diverse reference frames (space, time, object, and subject) is our fundamental ability used every day to make sense of the reality that surrounds us (see Goldstone and Barsalou, 1998; Sagiv et al., 2011).

## Swimming Style-Color Synesthesia or a New Variant of Mirror-Sensory Synesthesia. Future Directions for Interpreting the State of the Art in Synesthesia Research

**Figure 1:**
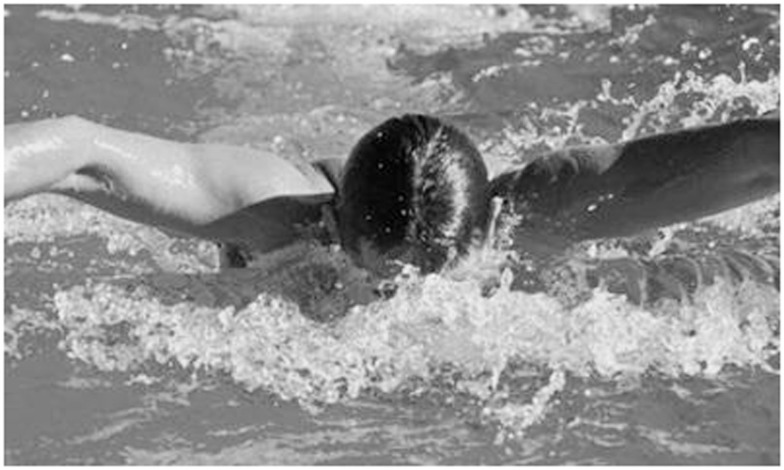
**An example photograph shown subjects in the study of Nikolić et al. (2011)**.

A novel form of synesthesia, swimming style-color synesthesia, has been discovered in two known grapheme-color synesthetes, who are semi-professional swimmers. The visual experience or imagination of four different swimming styles (breaststroke, butterfly, crawl, and backstroke) is synesthetically associated by them with four different colors (Nikolić et al., 2011). The induction of this kind of synesthesia took place exclusively under laboratory conditions and did not require any measurements in a swimming pool. All what subjects had to do was to take a look at photographs of other people swimming (e.g., Figure 1) or to think about/mentally visualize a given swimming style. This was sufficient to elicit their color experience.

**Figure 2:**
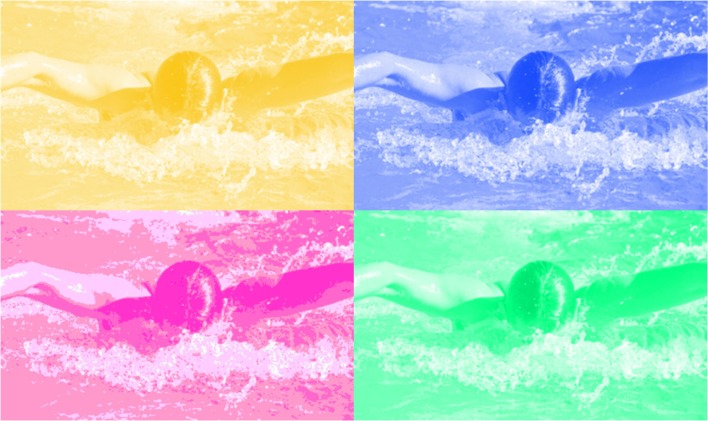
**Stimuli used in the Stroop test: Example pictures of a person swimming butterfly stroke, painted either in a subject’s synesthetic color (congruent) or in one of his non-synesthetic colors (incongruent)**. Reprint from Nikolić et al. (2011).

Not only subjects’ reports were taken into account, but also two standard tests for synesthesia, the consistency test, and the Stroop task, provided objective evidence for the existence of swimming style-color synesthesia (see Baron-Cohen et al., 1987; Odgaard et al., 1999; Mroczko et al., 2009). Swimming style-color synesthetes reported significantly more consistent colors than control non-synesthetes trained in these associations. Furthermore, in a Stroop task the color naming times accelerated when the color of the colored photograph was congruent with the synesthetic color of the presented swimming style and slowed down when the color was incongruent (Figure 2).

Hence, swimming style-color synesthesia is a genuine form of synesthesia similar to other common types of the phenomenon, such as grapheme-color synesthesia or colored hearing, although mostly related to mirror-sensory variants. As such swimming style-color synesthesia might be understood as mirror-movement associated with color experience. According to our hypothesis further brain imaging studies should reveal the respective activation of mirror systems after such multimodal, or even multi-domain, visuo-motor stimulation. Interestingly, the current findings demonstrate clearly that inducers of swimming style-color synesthesia do not have to be modality-dependent sensations directly; the activation of kinesthetic simulation does suffice. The mirror based embodied emulation of another’s action eliciting synesthetic concurrents can be interpreted in terms of cross-domain integration which fits well with the above mentioned cognitive account of synesthesia as an overall epistemic ability, *the unity of cognition* (Mroczko-Wąsowicz, in press). The original study showed that this kind of synesthesia may occur even when direct sensory or motor inputs are absent and only indirect ones exist. This allows us to conclude on the complex nature of the phenomenon. It is evidence for an extended case of embodied emulation in which the synesthetic concurrent is elicited without any direct motoric stimulation, only through kinesthetic emulation activated by the mental visualization of a certain swimming style. This means, the motor imagery of a certain swimming style seems to produce motor and somatosensory activations in form of the mirror system based motor emulations. The latter ones, based on the individual connections, lead to synesthetic color sensations. This form of synesthesia has some special features allowing the separation of direct sensory inputs, i.e., motor and proprioceptive inputs during the act of swimming, from those that evoke mental emulations, i.e., pictures, thoughts, or words related to respective swimming activities. Hence, the observation of a picture representing a swimming action may induce the creation of the sensory-motor emulation on a higher conceptual level. This again supports the possibility of a form of semantically driven synesthesia. Obviously, the fact that the synesthetes did not have to undergo any physical exercise does not imply that their motor system was entirely silent. Regardless whether it was a mirror based embodied simulation, a motor imagery based embodied emulation, or a result of the action concept activation, there is a motor neural representation of action even in the absence of any explicit motor behavior.

What enables the direct matching between the visual representation of an action, its motor representation, and further synesthetic color representation is still a matter of questions and speculations (see Rizzolatti et al., 2001). Early forms of automatic imitation of adult facial and manual gestures by human neonates (Meltzoff and Moore, 1977, 1983, 1994) seem to suggest some hard-wired mechanisms coupling action observation with action execution. The traditional view on synesthesia as a form of cross-wiring between senses, would suggest that the above described phenomenon could arise only by providing corresponding motor inputs directly. To the contrary, the referred study indicated that no direct motor or somatosensory stimulation is necessary. To validate experiences characteristic for synesthesia from the third person perspective, it was sufficient to activate the respective emulations by showing pictures of swimming persons. Therefore, we can conclude that visualization of a given swimming style, be it perceptual or imaginary may induce a corresponding synesthetic color experience in the absence of any overt muscular activation. On the basis of these results as well as of findings concerning the inducing role of imagined graphemes in grapheme-color synesthesia (Frith and Paulesu, 1997; Ramachandran and Hubbard, 2001a) and sensory-motor contingency in observing dancers of ballet and capoeira (Calvo-Merino et al., 2005, 2006) the following generalization to swimming style-color synesthesia seems to be legitimate: kinesthetic emulation triggers concurrent color sensations, much like the original motor input itself. If the activation of motor emulations is sufficient for the concurrent to be evoked, other cases of synesthesia and synesthesia-like processes may be revealed in which particular body movements serve as inducers, e.g., different styles and techniques in sports. This refers not only to motion and behavior. Since producing vicarious activations enables the observer of another person’s sensation, emotion, or action to feel as if he were experiencing that sensation, emotion, or performing that action himself, triggering synesthetic experiences may be mediated by the activation of a specific model that constitutes an internal, brain-based emulation of the perceived event, and of organism-environment interactions. The hypothesis can immediately be applied to the category of emotions – already known cases of emotion driven synesthesia (e.g., emotion to color synesthesia, personalities inducing colors, smells, or touch; see Ward, 2004; Sinha, 2009) might well be extended to inducers in form of observed emotions experienced by other people.

Also, distinctive features of swimming style-color synesthesia may explain why, in synesthetes, only some types of modality/domain related emulations produce synesthesia. Both synesthetes have been active swimmers since early childhood. This supports the hypothesis that at that time, when synesthesias are known to develop (Baron-Cohen et al., 1987; Harrison and Baron-Cohen, 1997), the categories of inducers that are especially disposed to acquire synesthesia are those that play a central role in the child’s life. Synesthesia seems to develop most easily for activities and emulations of them that children spend most time with and that possess predominant representational contents in the course of learning and playing. Therefore, theoretically there is no reason to exclude any kind of mental representation from being a possible inducer of concurrent synesthetic experiences. The key aspect seems to be the frequency with which this potential trigger is being employed by a young synesthete. The present results imply that the initiation of synesthetic associations may be regulated by the ability of eliciting emulations of regularly experienced events, properties, or situations.

## Hyperbinding, Emulative Semantics, and the Theory of Neuro-Frames

**Figure 3:**
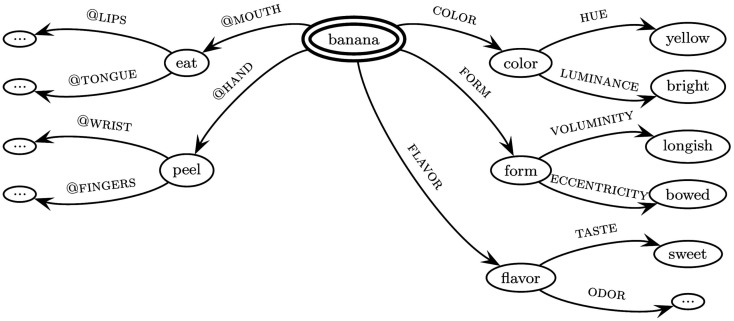
**Hypothetical fragment of the frame for the concept [banana]**. The substance concept to be decomposed is marked by a double-circle as the referring node of the frame. The labeled arrows denote attributes, the nodes their values. Nodes are themselves regarded as concepts and thus as conceptual parts of the central concept. In English, feature attributes (shown on the right) are frequently lexicalized – their arguments typically enter possessive constructions like *The color of the banana is yellow* or *The banana has the color yellow*. Based on linguistic and neurobiological evidence, we assume that affordances often relate to body parts and hence use the convention “@ + body part.” Formally, attributes are mappings from domains of some type into domains of some other type. Petersen and Werning (2007) provide an explicit account of frames using a calculus of typed feature hierarchies and incorporating typicality effects.

Swimming style-color synesthesia may also be viewed as a non-standard binding pattern between the neurobiologically realized attributes of color and bodily motion. Binding patterns between intra- and cross-modal as well as cross-domain attributes, including i.a. perceptual, proprioceptive, emotional, numerical, and motor attributes, in ordinary cases play an important role in the formation of concepts. As an underlying theoretical framework, the theory of neuro-frames (Werning and Maye, 2005, 2007; Werning, 2012) has been developed. This neuro-cognitive model of situated conceptualization (Barsalou, 2008) postulates neuro-frames as neuronal bases for concepts. A frame is defined for a large domain of things and contains a fixed set of attributes, each of which allows for a number of different values (Barsalou, 1992). The attributes in question are not constrained to perceptual modalities, but may involve motor attributes as well as further attributes (in this paper we are focusing on the interrelation between perceptual and motor attributes). Frames can be nested hierarchically and mutual constraints between attributes (e.g., between states of an object and actions directed to it) and between larger frames can be incorporated (see Figure 3).

For many attributes involved in perceptual processing one can anatomically identify cortical correlates. Those areas often exhibit a twofold topological structure and justify the notion of a feature map: (i) a receptor topology (e.g., retinotopy in vision, somatotopy in touch): neighboring regions of neurons code for neighboring regions of the receptor; and (ii) a feature topology: neighboring regions of neurons code for similar features. With respect to the monkey, more than 30 cortical areas forming feature maps are experimentally known for vision alone (Felleman and van Essen, 1991).

**Figure 4:**
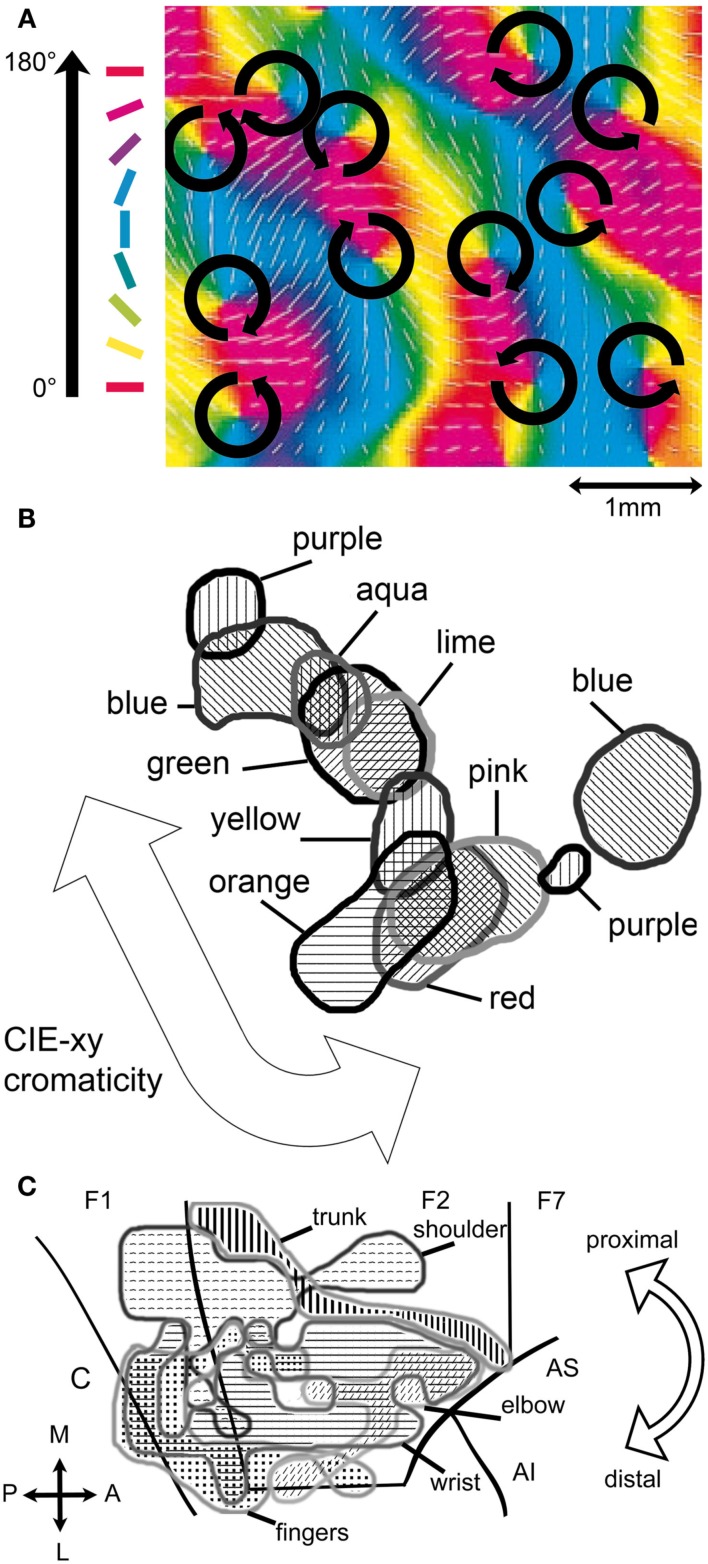
**Cortical realizations of frame attributes**. **(A)** Fragment of the neural feature map for the attribute orientation of cat V1 (adapted from Shmuel and Grinvald, 2000). The arrows indicate the polar topology of the orientation values represented within each hypercolumn. Hypercolumns are arranged in a retinotopic topology. **(B)** Color band (ca. 1 mm^2^) from the thin stripes of macaque V2 (adapted from Xiao et al., 2003). The values of the attribute color are arranged in a topology that follows the similarity of hue as defined by the Commission Internationale de l’Eclairages (*xy*-chromaticity). The topology among the various color bands of V2 is retinotopic. **(C)** Neural map (ca. 250 mm^2^) of forelimb movement in macaque primary motor (F1) and dorsal premotor cortex (F2, F7) (adapted from Raos et al., 2003). The overarching topology is somatotopic from proximal to distal movement as shown by the arrow. Due to the size of the region one may expect it to comprise maps for more specific motor attributes. C: central sulcus, AS, and AI: superior, respectively, inferior arcuate sulcus.

Motor attributes may also be parts of frames and appear to have cortical correlates, predominantly in the premotor and motor cortex (Werning, 2010). The cortical organization of motor control with regard to the effectors follows similar topological principles as the cortical organization in perception with regard to the receptors. The discovery of the so-called canonical motor neurons in the mirror neuron system, activated by the sight of an object to which a certain action is applicable (Rizzolatti and Luppino, 2001; Rizzolatti and Craighero, 2004), may provide a basis to integrate affordances (Gibson, 1977) – specific qualities of the object that allow the agent to perform particular actions upon it – into frames. Figure 4 shows a number of neural maps that relate to various attributes of frames.

Canonical neurons are involved in mechanisms for recognizing object affordances and contribute to the semantic knowledge about the object (Sahin and Erdogan, 2009). Hence, the activation of the mirror system brings its multimodal neurons to respond not only to action performance, but also to visual, auditory, somatosensory, and proprioceptive signals. This suggests that related processes are grounded functionally by multimodal circuits (Gallese and Lakoff, 2005; Rizzolatti and Sinigaglia, 2010). In particular, the intraparietal sulcus and inferior parietal lobule are involved in multisensory integration and vicarious sensory-motor activations (Bremmer et al., 2001; Rozzi et al., 2006; Ishida et al., 2010; Rizzolatti and Sinigaglia, 2010). These regions, able to receive visual input, are directly connected with each other and with the somatosensory cortex (i.e., BA 2; Pons and Kaas, 1986; Lewis and van Essen, 2000) integrating tactile and proprioceptive stimuli as well as containing shared sensory-motor representations (Keysers et al., 2010). These multimodal circuits exhibit some basic semantic features. The activation of a specific action concept, e.g., expressing an affordance or any other motor attribute, induces the activation of the multimodal neural circuits (Pulvermüller and Fadiga, 2010). In swimming style-color synesthesia the functioning of such a multimodal mechanism might take place when synesthetes swim, see other person swimming, when they see a swimming pool and recognize this object affordances or use a concrete action concept such as for example [breaststroke].

The fact that values of different attributes may be instantiated by the same object, but are processed in distinct regions of cortex is a version of the binding problem (Treisman, 1993; Tacca, 2010): how is this information integrated in an object-specific way? How can the color and taste of a banana be represented in distinct regions of cortex, although they are part of the representation of one and the same object? A prominent and experimentally well supported solution postulates oscillatory neural synchronization as a mechanism of binding: Clusters of neurons that are indicative of different properties sometimes show synchronous oscillatory activity, but only when the properties indicated are instantiated by the same object in the perceptual field; otherwise they are firing asynchronously. Synchronous oscillation, thus, might be regarded as fulfilling the task of binding various property representations together to form the representation of an object having these properties (Singer, 1999). Using oscillatory networks as biologically motivated models, it could be demonstrated how the topological organization of information in the cortex by mechanisms of synchronization may yield a logically structured semantics of concepts (Maye and Werning, 2004; Werning and Maye, 2007). Compositionality theorems have been provided (Werning, 2005). Oscillation functions play the role of object concepts. Clusters of feature sensitive neurons play the role of attributive concepts. The experimental findings by Schnitzler et al. (2006) on the essential role of neural synchronization for action control may justify the extension of the synchrony-based neuro-frame approach from features to affordances. It should be noted that the envisaged semantics is one of emulation: the neuronal structure is partially isomorphic to a (model-theoretic) model of the representational content. A concept like [banana] thus interrelates i.a. sensoric and motoric emulations: Having the concept [banana] means being able to emulate what a banana would look, taste, feel, and smell like and being able to emulate actions afforded by a banana. Triggering the concept activates the respective sensoric and motoric cerebral regions for the purpose of emulation even in the absence of a real banana. The neuro-frame captures how the various sensoric and motoric emulations are linked to each other. Emulative semantics is a non-symbolic, embodied, but still compositional semantics and might be used to link conceptual resources employed in perception and motor planning to linguistic meaning (Werning, 2012).

Support for the theory of neuro-frames also comes from a number of neuro-linguistic studies. Based on a review of neurobiological data, Pulvermüller et al. (1999) suggests that neural assemblies that pertain to the sensory-motor cortices and are bound by neural synchronization play an important role in understanding the meanings of words and sentences. These cortical sensory-motor action and perception circuits are interdependent in language comprehension. Neuroimaging investigations have shown that perception and understanding of stimuli depend on motor circuits, i.e., specific motor activations can be found when subjects understand speech sounds, word meanings, semantic categories, and sentence structures (Pulvermüller and Fadiga, 2010). FMRI studies (Pulvermüller, 2005) regarding the understanding of verbs, e.g., hint at a differential top-down activation of motor and pre-motors areas. We know that the understanding of concrete nouns like *hammer*, for which not only features, but also affordances are salient, results in an activity distributed over the premotor and the visual cortex (Martin et al., 1996; Martin, 2007). The hypothesis that words for substance concepts arouse more widely distributed activity than words for attributive concepts has also been supported by EEG studies (Rappelsberger et al., 2000). Brain areas involved in motor control contribute to neural networks in which verb representations are grounded, e.g., studies on motor deficits such as Parkinson disease reveal impairment of patients’ action naming (Rodríguez-Ferreiro et al., 2009). Higher-order abilities such as thinking or linguistic concept use are based in sensory-motor abilities.

Unlike in normal concept formation, where perceptual and motor attributes forming a concept are bound together into a frame (e.g., the concept [banana] in Figure 3), synesthesia may generally be regarded as a case of hyperbinding (Emrich et al., 2002, 2004; Sagiv and Robertson, 2005; Mroczko-Wąsowicz, in press). Attributes that do not form a sensible concept frame are bound together. This is especially striking in the case of visuo-motor or mirror-movement to color synesthesia as observed in swimming style-color synesthesia. Certain attributes of bodily motion are contingently linked to the attribute color. An additional synesthetic attribute of the concurrent is not only bound additively to the attributes of the inducer, it is experienced integratively. Also, one and the same attribute of the synesthetic inducer may be integrated with two values, the ordinary and the synesthetic one. E.g., in grapheme-color synesthesia the letter “A” is bound to the values “black” and “red” of the attribute color. A plausible hypothesis for the wide-ranging phenomenon of synesthesia thus might be that it results from a process of hyperbinding. If one assumes the neurobiological hypothesis that binding is achieved by some sort of neural synchronization between neurons that code for attribute values (perceptual features, motor affordances, etc.) then hyperbinding might be neurobiologically manifested by “too much” synchrony between “too many” neural feature maps and clusters in “too many” cortical regions. Alternative hypothesis of how binding is achieved might lead to analogous predictions.

## Conclusions

This paper undertakes the broader attempt of understanding the role of sensory-motor processes in synesthesia as part of a theory of mental representation as emulation. This regards the involvement of emulations in higher-level cognitive functions such as visual and motor imagery, object and action recognition, iconic memory, the representation of object and action concepts and language comprehension (Rumiati and Caramazza, 2005; Rumiati et al., 2010). The reconsideration of mental processes involved in synesthesia-like experiences proposed here will lead to vital implications not only for synesthesia research, but also generally for theories of perception and cognition. The wide spectrum of synesthesia-like processing extends itself from multimodal sensory or sensory-motor phenomena, through universal cross-modal or cross-domain correspondences to linguistic metaphors (Day, 1996; Martino and Marks, 2001; Sagiv and Ward, 2006), linking sensory-motor contingency and emulation with synesthetic associations, language comprehension, and social competencies. The interpersonal experience of mirror-sensory synesthesia is thought to rely on hyperactivity of the same brain mechanisms that we all use when observing another person’s experiences. It therefore offers insights into how our brain shapes interpersonal representations between self and others.

The comparison between different psychological phenomena employing synesthesia-like processing as well as the use of a peculiar type of synesthesia have the potential of challenging conventional thinking on this phenomenon and the existing interpretations. If swimming style as a kinesthetic simulation can induce synesthetic color perception, then interpretations in terms of bottom-up hard-wired structural connectivity, postulated not only between word/grapheme form and color areas in grapheme-color synesthesia, but generally in synesthesia research, may be challenged. Interpretations in terms of more top-down associations that are related to complex semantic representations might fit better.

## Conflict of Interest Statement

The authors declare that the research was conducted in the absence of any commercial or financial relationships that could be construed as a potential conflict of interest.
